# The burden of mortality due to injury in Cabo Verde, 2018

**DOI:** 10.1371/journal.pone.0278589

**Published:** 2023-03-13

**Authors:** Ngibo Mubeta Fernandes, Maria da Luz Lima Mendonça, Lara Ferrero Gomez

**Affiliations:** 1 National Public Health Institute, Praia, Cabo Verde; 2 Department of Natural, Life and Environmental Sciences, University Jean Piaget, Praia, Cabo Verde; Tehran University of Medical Sciences, ISLAMIC REPUBLIC OF IRAN

## Abstract

External causes continue to be one of the main causes of mortality in the world and Cabo Verde is no exception. Economic evaluations can be used to demonstrate the disease burden of public health problems such as injuries and external causes and support prioritization of interventions aimed at improving the health of the population. The objective of this study was to estimate the indirect costs of premature mortality in 2018 due to injuries and other consequences of external causes in Cabo Verde. Years of potential life lost, years of potential productive life lost and human capital approach were used to estimate the burden and indirect costs of premature mortality. In 2018, 244 deaths were registered due to injury and other consequences of external causes. Males were responsible for 85.4% and 87.73% of years of potential life lost and years of potential productive life lost, respectively. The cost of productivity lost due to premature death caused by injury was 4,580,225.91 USD. The social and economic burden due to trauma was substantial. There is a need for more evidence on the burden of disease due to injuries and their consequences, to support the implementation of targeted multi-sectoral strategies and policies for the prevention, management, and reduction of costs due to injuries in Cabo Verde.

## Introduction

The world loses nearly five million people annually to trauma, poisoning and other external causes, which corresponds to 9% of global deaths [1]. Injuries continue to have significant weight on the global disease burden, and are among the main causes of morbidity and mortality in adolescents, young people and adults [2]. In addition, injury represent a considerable public health problem, especially in developing countries, which are responsible for almost 90% of the world’s fatalities [3, 4]. Furthermore, the burden of injuries and external causes of morbidity and mortality has a considerable social and economic impact. For example, road accidents are among the main causes of death in young people and cause losses of up to 3% of the Gross Domestic Product (GDP) globally and up to 5% in low- and middle-income countries [5].

Despite being a considerable public health problem, injuries and their consequences were not prioritized on the world agenda [1, 6]. However, recognizing the magnitude of injuries and other consequences of external causes, goals have been set globally to reduce premature mortality rates from suicides and road accidents, and to strengthen prevention and treatment measures of important determinants of injuries and external causes by 2030 [5, 7].

Injuries and the consequences of external causes are a major burden of disease globally, moreover, they mainly occur in developing countries, where few studies evaluating their economic impact and burden of diseases have been carried out [6]. Economic analysis of the implementation of interventions to assess the results and costs of these interventions aimed at improving the health of the population are recommended [4, 8] and are used to demonstrate the disease burden in monetary terms [9–11].

Injuries continue to be one of the main causes of mortality and morbidity in the world, and Cabo Verde is no exception. In 2018, Cabo Verde recorded 2838 deaths, of which 244 were due to trauma, which represented a mortality rate of five (5) deaths per 1000 inhabitants and were the second leading cause of admissions in central hospitals in the country [12]. In addition, the country recognizes injuries and external causes as a public health problem that deserve the proper framework in the national health policy and a multisectoral approach to address this problem [13].

Cabo Verde is one of the Small Island Developing States (SIDS), and is located 450–500 km from the west coast of Africa and is characterized by scarce natural resources, recurrent droughts, with a relatively small market economy. Cabo Verde is a lower middle-income country with a tourism-based economy. Political stability and good governance have contributed to the economic success of the country [14]. The archipelago is made up of 10 islands and has a land surface of 4,033 square kilometers [15] and had an estimated population of 544,081 in 2018 [16].

The current study is the first carried out in Cabo Verde estimating the indirect costs of mortality and demonstrating the socioeconomic burden due to injuries and consequences of external causes.

## Materials and methods

### General approach

The study is a cross-sectional analysis, with descriptive analysis of the epidemiological profile of premature mortality due to injury and a partial economic evaluation of the costs of premature mortality, adopting the socioeconomic perspective. In the mortality data used, deaths from injuries were classified into two groups: external causes and injuries and consequences of external causes. For this study, and all deaths classified in both groups were considered.

### Source and data collection

The Integrated Surveillance and Response Service of the National Health Directorate of the Ministry of Health and Social Security (MSSS) made available mortality data for 2018. The data were anonymized, in order to guarantee the confidentiality of individual data. Deaths were filtered by age, sex, municipality and cause of death. All deaths classified with codes S00-T98 and V01-Y98 of the International Classification of Diseases and Related Health Problems, Tenth Revision (ICD-10) were extracted for analysis [17].

Descriptive data analysis was performed using Microsoft Excel 2016 and Statistical Package for Social Science (SPSS, version 22) and Mendeley Reference Management Software [18], was used to organize the bibliographic references.

### Estimation methods

Life expectancy and the human capital approach were used to estimate the burden and costs of premature mortality. The human capital approach was applied to estimate the value of potential lost productivity due to mortality due to injury [19]. To estimate the disease burden of premature mortality, measures of years of potential life lost (YPLL), years of potential productive life lost (YPPLL) and cost of productivity lost CPL were used [9, 10, 19, 20]. These concepts estimate the average time a person would live if they did not die prematurely, taking into account the age limit (death/retirement) and the cause [9]. YPLL estimates were based on the average value of life expectancy at birth in 2018 [21].

The YPLL were determined using the formula:

$$YPLL=\sum_{i=I}^{I}a_{i}d_{i}$$ (1)

Where: *a*_*i*_ = (73 −i−0.5) the number of years left to the stipulated age limit, *d_i_* = the number of deaths in the age between i and i + 1.

YPLL rates were stratified by municipality, sex, age group and cause of death. Demographic projections [16] were used to calculate the YPLL rates.


$$Rate\;of\;YPLL=\sum_{i=I}^{I}a_{i}d_{i}\times \frac{10^{n}}{N}$$ (2)

Where: N = number of people between 1 and 72 years of age in the population. Note: ai and di are already defined above.

To estimate the YPPLL, the age groups from 15 to 64 years old constituted the national workforce [22].


$${YPPLL}_{j}=\sum_{j=1}^{J}d_{j}\times W_{j}$$ (3)

Where: *d_j_* = average number of deaths for each age group W_*j*_ = midpoint for retirement age in each age group = 65-*j*-0.5 and *j* = are groups of 5 years for the entire productive population (15–64).

The YPPLL and the Gross Domestic Product (GDP) *per capita* of 3617.33 USD for the year 2018 [21], were used to estimate the CPL due to premature mortality using the following formula:

$$CPL=\sum_{j=1}^{J}\left(YPPLL\right)\times GDP\;per\;capita$$ (4)

For this study it was assumed that the individual’s productivity did not change until retirement [9]. The present values of future costs were calculated by applying a discount rate of 4.5% practiced by the Bank of Cabo Verde [23]. In addition, a univariate sensitivity analysis was performed considering alternative discount rates (3% and 6%) to determine how different discount rate values would affect present productivity costs [8, 24].

The present value was obtained using the following formula:

$$Present\;value={CPL}_{j}\sum_{j=1}^{J}\frac{1}{{\left(1+r\right)}^{t}}$$ (5)

Where: *CPL* = future costs for each age group, *r* = discount rate, *t* = average point to reach retirement age for each age group and *j* = are groups of 5 years for the entire productive population (15–64).

### Ethical and legal considerations

This study was approved by the National Ethics Committee for Health Research (*CNEPS*) through resolution n° 33/2020 of May 28, 2020 and the National Commission for Data Protection (*CNPD*) through dispatch n° 183/2020. Being a retrospective study, no informed consent was required.

## Results

### Mortality profile

Cabo Verde recorded 244 deaths from injuries and external causes in 2018. Males were responsible for 84.4% (206) of deaths and around 23.3% (48) of deaths were among individuals with elementary occupations. Overall, age groups with the highest proportion of deaths were those between 20 to 24, 25 to 29 and 35 to 39, which represent 34.4% (84) of the cases registered in 2018 S1A Table in S1 File. Overall, the principal causes of death, were due to traumatic head injuries (23.0%; 56) and intentional self-harm (22.5%; 55). The main causes of death among females were assaults (21.1%; 8), followed by head injuries (18.4%; 7) and intentionally self-inflicted injuries which accounted for 15.8% (6) of deaths. S2B Table in S1 File.

### Years of potential life lost

Of the 244 deaths reported due to trauma, 211 (86.5%) were in individuals aged between 1 and 72 years old and these accounted for 7222 YPLL and on average, 34.2 YPLL were lost per death. Males lost on average of 33.7 YPLL and females lost 37.6 YPLL per death. Overall, the age range from 20 to 39 years, accounted for 60.3% or 4357 YPLL, and the same age group in the male population, accounted for 63.3% or 3906 of the YPLL. For females, the age group 1 to 4 years contributed 20% (210 YPLL) of the total YPLL in this group, compared to 1.9% (140) for males. Approximately 14 years were lost per 1000 inhabitants between 1 and 72 in 2018. Males and females had rates of 23.6 and 4.2 YPLL per 1000 inhabitants, respectively Table 1.

**Table 1:** Years of potential life lost, proportions, means and rates per 1000 inhabitants by sex and age group, Cabo Verde, 2018.

Age group	Male			Female			Total		
	YPLL	%	YPLL rate	YPLL	%	YPLL rate	YPLL	%	YPLL rate
1 to 4	140	1.9	6.5	210	2.9	10.3	350	4.8	8.4
5 to 9	262	3.6	10.4	66	0.9	2.7	328	4.5	6.6
10 to 14	242	3.4	9.4	0.0	0.0	0.0	242	3.4	4.7
15 to 19	111	1.5	4.6	111	1.5	4.7	222	3.1	4.6
20 to 24	1212	16.8	47.5	152	2.1	6.2	1364	18.9	27.3
25 to 29	865	12.0	30.1	228	3.2	8.7	1092	15.1	19.9
30 to 34	729	10.1	27.1	0.0	0.0	0.0	729	10.1	14.5
35 to 39	1101	15.2	50.0	71	1.0	3.9	1172	16.2	29.0
40 to 44	610	8.4	36.7	31	0.4	2.1	641	8.9	20.5
45 to 49	230	3.2	17.1	77	1.1	6.0	306	4.2	11.7
50 to 54	246	3.4	20.4	21	0.3	1.6	267	3.7	10.7
55 to 59	279	3.9	29.5	47	0.6	4.3	326	4.5	16.1
60 to 64	105	1.5	18.4	42	0.6	5.1	147	2.0	10.5
65 to 69	33	0.5	10.2	0	0.0	0.0	33	0.5	4.0
70 to 72	6	0.1	5.2	0	0.0	0.0	6	0.1	2.1
**Total**	**6170**	**85.4**	**23.6**	**1053**	**14.6**	**4.2**	**7222**	**100**	**14.0**

### The geographic distribution of YPLL

The distribution of YPLL due to injury varied between regions. The municipality of Praia contributed to the highest proportion of YPLL with 27.4% (1978), followed by the island of São Vicente with 982 (13.6%) and Santa Catarina with 782 (10.8%). The municipality of Brava had the highest rate of 34.5 YPLL per 1000 inhabitants and the lowest rate was recorded in the municipality of São Lourenço dos Órgãos with 5.5 YPLL per 1000 inhabitants. The municipalities of São Domingos and Ribeira Grande Santiago revealed the highest averages of losses per death of 46.6 and 45.5 YPLL and victims died on average, at 26.4 and 27.5 years, respectively. The municipality of Santa Catarina de Fogo had the lowest mean per death of 18.8 YPLL and, subsequently, the highest mean per death, of 54.2 years. The mean age at death from external causes in 2018 was 38.8 years Table 2.

**Table 2:** Years of potential life lost, proportions, means and rates per 1000 inhabitants by municipalities, Cabo Verde, 2018.

Municipality	YPLL	%	YPLL rate	Mean	Mean age at death
Brava	178	2.5	34.5	29.7	43.3
Paul	147	2.0	28.3	36.8	36.3
Tarrafal de São Nicolau	122	1.7	25.3	30.5	42.5
Ribeira Brava	152	2.1	24.0	38.0	35.0
Porto Novo	377	5.2	23.8	29.0	44.0
Tarrafal	346	4.8	20.3	34.6	38.4
Santa Catarina	782	10.8	17.9	34.0	39.0
São Salvador do Mundo	132	1.8	16.5	43.8	29.2
Boavista	248	3.4	14.5	41.3	31.8
Ribeira Grande	204	2.8	14.0	29.1	43.9
São Domingos	187	2.6	14.0	46.6	26.4
São Miguel	177	2.5	13.4	44.3	28.8
Praia	1978	27.4	12.7	42.1	30.9
São Vincent	982	13.6	12.5	30.7	42.3
Ribeira Grande de Santiago	91	1.3	11.4	45.5	27.5
Santa Catarina do Fogo	57	0.8	11.4	18.8	54.2
Maio	76	1.1	11.2	38.0	35.0
São Felipe	219	3	11.2	27.4	45.6
Sal	368	5.1	10.0	28.3	44.7
Santa Cruz	188	2.6	7.6	31.3	41.7
Mosteiros	66	0.9	7.5	33.0	40.0
São Lourenço dos Órgãos	36	0.5	5.5	35.5	37.5
Others	114	1.6	-	16.2	56.8
**Cabo Verde**	**7222**	**100**	**14.0**	**34.2**	**38.8**

### Years of potential life lost by cause of death

Causes due to intentional self-harm contributed the largest proportion of the total YPLL (25.8%; 1862) and similarly, for males and was responsible for 26.6%(1639). The largest contributor of YPLL for females were deaths from assault and accounted for 29.4% (309) of total YPLL among females. Regarding the mean YPLL per death, deaths from toxic effects of substances of essentially non-medicinal origin had the highest mean of 53.5 YPLL for both sexes and the same group of causes had the highest mean among males, 45 YPLL/death while for females, the highest average was 70 YPLL per deaths in the groups “causes from other effects and unspecified effects of external causes and toxic effects of substances of essentially non-medicinal origin” Table 3.

**Table 3:** Years of potential life lost, proportions and means by causes, Cabo Verde, 2018.

Causes of death	Male			Female			Total		
	YPLL	%	Mean	YPLL	%	Mean	YPLL	%	Mean
Intentional self-harm	1639	26.6	36	223	21.2	37	1862	25.8	36
Injuries to the head	1137	18.4	28	132	12.5	33	1268	17.6	28
Injuries involving multiple body regions	1056	17.1	36	168	15.9	34	1223	16.9	36
Accidental drowning and submersion	841	13.6	38	0	0.0	0	841	11.6	38
Assault	838	13.6	36	309	29.4	39	1147	15.9	37
Certain early complications of trauma not classified elsewhere	158	2.6	32	0	0.0	0	158	2.2	32
Other and unspecified effects of external causes	137	2.2	34	70	6.7	70	207	2.9	41
Other accidental threats to breathing	117	1.9	29	0	0.0	0	117	1.6	29
Toxic effects of substances of chiefly non-medicinal origin	91	1.5	45	70	6.7	70	161	2.2	54
Burns and corrosions	46	0.7	23	82	7.7	27	128	1.8	26
Injuries to unspecified part of trunk, limb, or body region	41	0.7	41	0	0.0	0	41	0.6	41
Injuries to the abdomen, lower back, lumbar spine and pelvis	36	0.6	36	0	0.0	0	36	0.5	36
Injuries to the neck	21	0.3	11	0	0.0	0	21	0.3	11
Poisoning by drugs, medicaments and biological substances	16	0.3	16	0	0.0	0	16	0.2	16
Injuries to the hip and thigh	0	0.0	0	0	0.0	0	0	0.0	0
**Total**	**6170**	**100**	**34**	**1053**	**100**	**38**	**7222**	**100**	**34**

### Years of potential productive life lost

Overall, 187 deaths occurred in individuals aged between 15 to 64 years and resulted in 4768 YPPLL. Males and females, accounted for 87.7% (4183) and 12.3% (585) of total YPPLL, respectively. The highest proportions of YPPLL were observed in the age group 20 to 24 years and similarly for males and corresponded to 24.1% (1148), 24.4% (1020), respectively, while for females, the highest proportion of YPPLL was observed in the age group from 25 to 29 years (32.1%; 188). For both sexes, the YPPLL rate was 13.2 per 1000 inhabitants, 23 per 1000 inhabitants for males and 3 per 1000 inhabitants of the female population Table 4.

**Table 4:** Years of potential productive life lost, proportions, means and rates per 1000 inhabitants by sex and age group, Cabo Verde, 2018.

Age group	Male			Female			Total		
	YPPLL	%	YPPLL rate	YPPLL	%	YPPLL rate	YPPLL	%	YPPLL rate
15 to 19	95	2.3	3.9	95	16.2	4.0	190	4	4.0
20 to 24	1020	24.4	40.0	128	21.9	5.2	1148	24.1	22.9
25 to 29	713	17.0	24.8	188	32.1	7.2	900	18.9	16.4
30 to 34	585	14.0	21.8	0	0.0	0.0	585	12.3	11.6
35 to 39	853	20.4	38.7	55	9.4	3.0	908	19	22.5
40 to 44	450	10.8	27.1	23	3.9	1.5	473	9.9	15.1
45 to 49	158	3.8	11.8	53	9.1	4.1	210	4.4	8.0
50 to 54	150	3.6	12.4	13	2.2	1.0	163	3.4	6.5
55 to 59	135	3.2	14.3	23	3.9	2.1	158	3.3	7.8
60 to 64	25	0.6	4.4	10	1.7	1.2	35	0.7	2.5
**Total**	**4183**	**100**	**23.0**	**585**	**100**	**3.0**	**4768**	**100**	**13.2**

### The geographic distribution of YPPLL

A total of 4768 YPPLL were calculated due to injuries and external causes, the municipality of Praia had the highest proportion of YPPLL (28.9%;1380), followed by the island of São Vicente (12.9%;615) and the municipality of Santa Catarina (10.2%;485). The geographic distribution of YPPLL rates was higher in the municipality of Brava with 37.9 per 1000 inhabitants, followed by the municipalities of the island of São Nicolau with 27.5 (Tarrafal) and 26.8 (Ribeira Brava). The municipalities of Ribeira Grande de Santiago and São Miguel recorded the highest losses, on average, of 37.5 and 36.3 YPPLL per death, respectively Table 5.

**Table 5:** Years of potential productive life lost, proportions, means and rates per 1000 inhabitants by municipalities, Cabo Verde, 2018.

Municipality	YPPLL	%	Rate	Mean	Mean age at death
Boavista	138	2.9	10.9	27.5	38
Brava	130	2.7	37.9	21.7	43
Maia	8	0.2	1.6	7.5	58
Mosteiros	50	1.0	8.7	25.0	40
Others	58	1.2	0.2	8.2	57
Paul	63	1.3	17.6	20.8	44
Porto Novo	278	5.8	25.3	25.2	40
Praia	1380	28.9	12.5	32.9	32
Ribeira Brava	120	2.5	26.8	30.0	35
Ribeira Grande	148	3.1	14.7	21.1	44
Ribeira Grande de Santiago	75	1.6	13.8	37.5	28
Sal	273	5.7	10.5	24.8	40
Santa Catarina	485	10.2	15.9	24.3	41
Santa Catarina do Fogo	33	0.7	10.3	10.8	54
Santa Cruz	140	2.9	8.6	23.3	42
São Domingos	93	1.9	10.4	30.8	34
São Filipe	158	3.3	12.3	22.5	43
São Lourenço dos Órgãos	28	0.6	6.2	27.5	38
São Miguel	145	3.0	16.4	36.3	29
São Salvador do Mundo	50	1.0	9.4	25.0	40
São Vincente	615	12.9	10.7	22.0	43
Tarrafal	215	4.5	18.8	26.9	38
Tarrafal de São Nicolau	90	1.9	27.5	22.5	43
**Cabo Verde**	**4768**	**100**	**13.2**	**25.5**	**40**

### Years of potential productive life lost by cause of death

Concerning the specific causes of death, for both sexes, intentional self-harm contributed 30.6% of YPPLL, followed by assaults with 19%, injuries involving multiple body regions with 17% and head injuries with 14%. On average, fatal victim of assaults recorded the highest mean YPPLL of 31, 32 and 31 for both sexes, for males and females, respectively. The overall mean YPPLL per death was 25, 26 YPPLL per death for males and 24 YPPLL for females. On average, males died at 39.3 years and females at 40.6 years Table 6.

**Table 6:** Years of potential productive life lost, proportions and means by causes, Cabo Verde, 2018.

Causes of death	Male			Female			Total		
	YPPLL	%	Mean	YPPLL	%	Mean	YPPLL	%	Mean
Intentional self-harm	1283	30.7	30	175	29.9	29	1458	30.6	30
Assault	663	15.8	32	245	41.9	31	908	19.0	31
Injuries involving multiple body regions	725	17.3	28	70	12	18	795	16.7	27
Injuries to the head	655	15.7	19	38	6.4	13	693	14.5	19
Accidental drowning and submersion	435	10.4	24	0	0.0	0	435	9.1	24
Certain early complications of trauma not classified elsewhere	118	2.8	24	0	0.0	0	118	2.5	24
Other and unspecified effects of external causes	105	2.5	26	0	0.0	0	105	2.2	26
Burns and Corrosions	30	0.7	15	58	9.8	19	88	1.8	18
Other accidental threats to breathing	85	2.0	21	0	0.0	0	85	1.8	21
injuries to unspecified part of trunk, limb, or body region	33	0.8	33	0	0.0	0	33	0.7	33
Injuries to the abdomen, lower back, lumbar spine and pelvis	28	0.7	28	0	0.0	0	28	0.6	28
Toxic effects of substances of chiefly non-medicinal origin	13	0.3	13	0	0.0	0	13	0.3	13
Poisoning by drugs, medicaments and biological substances	8	0.2	8	0	0.0	0	8	0.2	8
Injuries to the neck	5	0.1	3	0	0.0	0	5	0.1	3
Injuries to the hip and thigh	0	0	0	0	0.0	0	0	0.0	0
**Total**	**4183**	**100**	**26**	**585**	**100**	**24**	**4768**	**100**	**25**

### Estimated costs of productivity lost

The estimated cost of productivity lost due to premature death attributable to injuries and external causes was 4,580,225.91 USD. Males contributed 87.7% (4,053,833.79 USD) of the total CPL in 2018. The average CPL per death was 24,493.19 USD for both sexes, 24,870.15 USD for males and 21,933.01 USD for females. Approximately 21% (957,146.98 USD) of the total CPL was attributed to individuals between 35 to 39 years for both sexes, and 22%; (899,138.07 USD), for males, while for females, the highest proportion of CPL (24.2%; 127,341.62 USD) was observed in the 25–29 age group Table 7.

**Table 7:** Estimated costs of lost productivity by sex, age group and cause at 4.5%, Cabo Verde, 2018.

	Variables	Male			Female			Total		
		Present value	%	Mean	Present value	%	Mean	Present value	%	Mean
**Age group**	15 to 19	41546.06	1.0	20773.03	41546.06	7.9	20773.03	83092.11	1.8	20773.03
	20 to 24	555888.66	13.7	23162.03	69486.08	13.2	23162.03	625374.74	13.7	23162.03
	25 to 29	483898.15	11.9	25468.32	127341.62	24.2	25468.32	611239.77	13.3	25468.32
	30 to 34	495115.37	12.2	27506.41	0.00	0.0	0.00	495115.37	10.8	27506.41
	35 to 39	899138.07	22.2	29004.45	58008.91	11.0	29004.45	957146.98	20.9	29004.45
	40 to 44	591460.80	14.6	29573.04	29573.04	5.6	29573.04	621033.84	13.6	29573.04
	45 to 49	257973.72	6.4	28663.75	85991.24	16.3	28663.75	343964.96	7.5	28663.75
	50 to 54	306173.51	7.6	25514.46	25514.46	4.8	25514.46	331687.97	7.2	25514.46
	55 to 59	343393.11	8.5	19077.39	57232.18	10.9	19077.39	400625.29	8.7	19077.39
	60 to 64	79246.35	2.0	7924.63	31698.54	6.0	7924.63	110944.89	2.4	7924.63
	Total	**4053833.79**	**100**	**24870.15**	**526392.13**	**100**	**21933.01**	**4580225.91**	**100**	**24493.19**
**Causes of death**	Injuries to the head	806706.02	19.9	23726.65	56006.48	10.6	18668.83	862712.50	18.8	23316.55
	Injuries to the neck	15849.27	0.4	7924.63	0.00	0.0	0.00	15849.27	0.3	7924.63
	Injuries to the abdomen, lower back, lumbar spine and pelvis	29004.45	0.7	29004.45	0.00	0.0	0.00	29004.45	0.6	29004.45
	Injuries to the hip and thigh	0.00	0.0	0.00	0.00	0.0	0.00	0.00	0.0	0.00
	Injuries involving multiple body regions	658778.30	16.3	25337.63	82043.39	15.6	20510.85	740821.69	16.2	24694.06
	Injuries to unspecified part of trunk, limb, or body region	27506.41	0.7	27506.41	0.00	0.0	0.00	27506.41	0.6	27506.41
	Burns and corrosions	48650.43	1.2	24325.22	47775.06	9.1	15925.02	96425.49	2.1	19285.10
	Poisoning by drugs, medicaments and biological substances	19077.39	0.5	19077.39	0.00	0.0	0.00	19077.39	0.4	19077.39
	Toxic effects of substances of chiefly non-medicinal origin	25514.46	0.6	25514.46	0.00	0.0	0.00	25514.46	0.6	25514.46
	Other and unspecified effects of external causes	101056.58	2.5	25264.15	0.00	0.0	0.00	101056.58	2.2	25264.15
	Certain early complications of trauma not classified elsewhere	126571.04	3.1	25314.21	0.00	0.0	0.00	126571.04	2.8	25314.21
	Accidental drowning and submersion	413219.68	10.2	22956.65	0.00	0.0	0.00	413219.68	9.0	22956.65
	Other accidental threats to breathing	112187.11	2.8	28046.78	0.00	0.0	0.00	112187.11	2.4	28046.78
	Intentional self-harm	1108586.12	27.3	25781.07	152245.33	28.9	25374.22	1260831.45	27.5	25731.25
	Assault	561126.51	13.8	26720.31	188321.86	35.8	23540.23	749448.37	16.4	25843.05
	**Total**	**4053833.79**	**100**	**24870.15**	**526392.13**	**100**	**21933.01**	**4580225.91**	**100**	**24493.19**

The principal drivers of CPL by specific causes were intentional self-harm, which was responsible for 27.5% (1,260,831.45 USD) of the total losses, followed by head injuries that contributed for 18.8% (862,712.50 USD). Furthermore, injuries to the abdomen, back, lumbar spine and pelvis had the highest mean loss per death, amounting to 29,004.45 USD. The same cause had the highest mean among males of 29,004.45 USD, while for females, the highest mean per death was attributed to the group intentionally self-inflicted injury in the amount of 25,374.22 USD followed by the cause “aggression” in the amount of 23,540.23 USD per death Table 7.

A univariate sensitivity analysis was conducted to assess the effects of varying the discount rates. The analysis, at 3% was worth the total CPL 6,900,400.84 USD and 3,140,890.80 USD at 6%, with an average CPP per death of 36,900.54 USD and 16,796.21 USD, respectively. At 3%, the highest proportion of the loss which corresponded to 20.8% (1,434,803.65 USD) of the total CPL, was observed in the 35 to 39 age group and the highest proportion of loss in the amount of 1,976,502.46 USD was attributed to the “intentional self-harm” group. At 6%, proportions of CPL by age group and cause did not differ significantly, and the total CPL value was approximately 50% lower than the total CPP value at 3% S3C and S4D Tables in S1 File.

## Discussion

This study demonstrated the socioeconomic burden due to injuries and external causes in 2018. The results of the study showed that mortality from injuries and consequences of external causes affected males disproportionately. Similar studies have shown significantly high mortality rates in males [25–27] and these inequalities between the sexes may be associated with risky behavior, the influence of society on men’s behavior (masculinity) and in certain cases risky behavior is related to socioeconomic factors [28, 29]. In this study, the mean age of mortality due to trauma was 39 years and the most affected age groups were between 20 and 44 years. The findings of this study were similar to those reported in similar studies where the active population accounted for the largest proportion of fatal victims due to injury [26, 28].

The main causes of mortality by type of injury were traumatic head injuries followed by traumatic injuries involving multiple body regions. These results collaborate findings showing that head injuries were among the principal types of trauma that lead to death in patients [19, 30]. When considering only injuries with chapter XX codes, it was observed that self-harm, assault, and drowning were the predominate causes of death. In comparison Nobre *et al*. and Pillay-van Wyk *et al*. [31, 32] reported aggressions and traffic accidents as the main causes of death and while Malta *et al*. and Xing *et al*. [33, 34] reported traffic accidents and suicides as the principal external causes of death and finally, Corassa *et al*. [35] indicated to suicides and aggressions as the most prevalent causes. The prevalence of suicide has been associated with mental disorders and sociodemographic characteristics and aggression with socioeconomic inequalities, alcohol and drug-related harm, respectively [26, 33]. In addition, traffic accidents were among the leading causes of death in Sub-Saharan Africa and while globally, traffic accidents, falls and interpersonal violence were identified as the leading causes of death due to injury [25, 36].

In this analysis, traffic accidents were not among the main causes of death in 2018. This was supported by data from other trauma surveillance systems that showed a low number of deaths due to traffic accidents [37]. Comprehensive road safety laws and their proper enforcement could contribute to the reduction of mortality from road accidents [25, 36]. Cabo Verde had approved a highway code in 2007 which has brought substantial gains in road safety [38].

In this study, assault was among the leading causes of death in the sample population and the principal cause among females. Cabo Verde has recognized gender-based violence as a social and public health problem and subsequently approved laws on the issue [39] and recognized that multiple socio-economic and cultural factors allow the existence of this phenomenon [40]. Furthermore, gender-based violence accounted for 20% of crimes reported [37] thus, supporting the results of the current study. Insufficiency in the implementation and regulation of laws could explain the prevalence of gender based violence [35]. Overall, mortality due to violence was prevalent in males than in females and has also been attributed to socioeconomic factors and other heath determinants [28, 29].

In the current analysis, drownings accounted for approximately 10% of mortality in the sample population, and 95.8% of these deaths were males. In Seychelles, drowning was the leading cause of death due to external causes [41]. The author associated this with the fishing industry, one of the country’s main economic and leisure activities and the need to strengthen safety measures in these industries. Despite being a small island country, similar to Cabo Verde, Abio *et al*. [41] suggested the promotion of swimming programs as a measure to reduce the risk of drowning in the population. Drownings occurred mainly in adult males, however approximately 20% of deaths were in children in this analysis and drowning has been highlighted as one of the leading causes of death in children [35, 42].

The estimated YPLL corresponded to a total of 7,222 and, a mean of 34.2 YPLL per death, which was higher than the mean obtained in Tanzania [9] and in line with the findings from Brazil which showed that males were responsible for the highest proportion of calculated YPLL, while the age ranges from 20 to 39 were responsible for approximately 60% of YPLL [35]. The differences in the mean and prevalent age groups could explain the difference in life expectancy used to obtain YPLL and other socioeconomic factors. Similar to the current study, males contributed to a higher proportion of YPLL due to injuries, and overall injuries affected the potentially productive population [10, 35, 43]. Among the major contributors to YPLL were deaths due to self-harm, assault and drowning, this result corroborates findings by Delgado [44].

Deaths among individuals between 15 and 64 years of age resulted in 4768 YPPLL, on average 25 YPPLL per death. Intentional self-harm and aggression were responsible for approximately 50% of YPLL and YPPLL and suicides were responsible for 30% of YPPLL. Corassa *et al*. [35] and Rajabali *et al*. [45] demonstrated that a significant proportion of YPPLL was attributable to self-harm in their studies. Additionally, injuries and other consequences from external causes accounted for 10% of lost productivity in the WHO African region, with self-harm and interpersonal violence accounting for 87.2% of productivity losses due to intentional injuries [46]. In economic terms, YPLL and YPPLL in individuals aged 20 years presented the greatest socioeconomic loss because this age corresponds to the age at which the greatest investment has been made by society and the beginning of the return on that investment to society [47].

In this study, the total cost of lost productivity due to premature death from injuries and external causes was 4,580,225.91 USD, men contributed 87.7% of the losses and overall each death resulted in losses amounting to 24,493.19 USD. In other studies, CPL due to injury was significantly higher among men than among women [10, 48]. Najafi *et al*. [10] reported that the highest average loss per death was due to trauma, amounting to 72,571 USD/death while Davey *et al*. [48] presented a mean value of approximately 2500 USD per death. A study conducted in a high-income country estimated values of lost productivity almost three times higher than the estimates found in this study [45]. Variations could be attributed to differences in the age limits, study periods, the GDP per capita and economic factors related to the countries in which the studies were conducted.

The present value of the CPL at 4.5% discount rate corresponded to approximately 0.2% of GDP in 2018, considering that GDP was estimated at 1.967 billion USD. This was lower than estimates of the economic burden due to road traffic accidents in low- and middle-income countries [5].

In the database used for this study, 47.1% (115) of the cases, deaths were classified by type of lesion, therefore the external causes that led to the appearance of the lesion were unknown, and 52.9% (129) were classified by external causes. ICD-10 guidelines recommended the use of codes from chapter XIX and XX to classify traumatic events and if only one code is used, chapter XX should be prioritized [17]. Results of this study demonstrate the need to standardize the classification of injuries and this could improve current estimates of mortality from external causes and complement national and global disease burden estimates [7, 26]. Standardization can be achieved through regular training of health professionals on current guidelines [41] and the digitization of health information systems.

Available data on economic costs of diseases, shows that two main methods are used to evaluate the economic impact, namely the friction method and the human capital method, with the human capital method being the most used methodology in cost-of-disease analyses [49]. It is argued that the friction method may result more accurate estimates of lost productivity losses when compared to estimates found using the human capital method [50]. For future studies, both methods maybe applied to better evaluate the economic impact of premature mortality.

### Limitations

Data analysis confirmed the incomplete classification of causes of death due to injury. This did not allow for analysis to be done exclusively by external causes or by the anatomical site of the lesion. The approach chosen made it possible to analyze all deaths recorded due to trauma, however it made it difficult to compare with other studies on the subject. This study demonstrated the need for standardization and training in the use of recommended tools for classifying injuries.

The methodology adopted did not include analysis of potential loses in individuals less than 1 and older than 72 years which could contribute to underestimation of the burden of injury. Analyzing the results presented in this study, the aforementioned limitations must be taken into account.

## Conclusion

The study demonstrated the socio-economic burden on the healthcare system and society due to injuries and showed that the costs of lost productivity due to premature mortality were substantial. The analysis showed that males were disproportionately affected by injuries. In addition, the largest proportion of deaths due to injuries was in young people with the potential to contribute to the socio-economic development of the country. Attention was drawn to the prevalence and substantial monetary burden of self-harm, drowning and violence in the population sample. Finally, the results of this study could be used to assess the need for implementation of other public health policies concerning injuries, and targeted strategies for injury prevention.

## Supporting information

S1 File (RAR)

## References

[1] World Health Organization. INJURIES VIOLENCE THE FACTS The magnitude and causes of injuries. Geneva World Heal Organ [Internet]. 2014;20. Available from: http://www.who.int/violence_injury_prevention/media/news/2015/Injury_violence_facts_2014/en/.

[2] Abbafati C, Abbas KM, Abbasi-Kangevari M, Abd-Allah F, Abdelalim A, Abdollahi M, et al. Global burden of 369 diseases and injuries in 204 countries and territories, 1990–2019: a systematic analysis for the Global Burden of Disease Study 2019. Lancet [Internet]. 2020;396(10258):1204–22. Available from: https://www.thelancet.com/pdfs/journals/lancet/PIIS0140-6736(20)30925-9.pdf. doi: 10.1016/S0140-6736(20)30925-9 33069326 PMC7567026

[3] Alberdi F, García I, Atutxa L, Zabarte M, Work NC, Care I, et al. UPDATE: AN UPDATE IN CRITICAL TRAUMA DISEASE Epidemiology of severe trauma ଝ. Med Intensiva (English Ed [Internet]. 2017;38(9):580–8. Available from: 10.1016/j.medine.2014.06.002. 25241267

[4] The World Bank. Disease Control Priorities in Developing Countries. In: Jamison DT, Breman JG, Measham AR, Alleyne G, Claeson M, Evans DB, et al., editors. Disease Control Priorities in Developing Countries [Internet]. second edi. New York and Washington DC: Oxford University Press and The World Bank; 2006. p. 737–53, 755–70. Available from: http://dcp-3.org/dcp2. 21250309

[5] Organização Mundial da Saúde. Escritório Regional para a África. Segurança rodoviária na região africana 2015 [Internet]. Brazaville; 2016. Available from: http://www.who.int/iris/handle/10665/246184.

[6] Wesson HKH, Boikhutso N, Bachani AM, Hofman KJ, Hyder AA. The cost of injury and trauma care in low-and middle-income countries: A review of economic evidence. Health Policy Plan [Internet]. 2014 Sep 1 [cited 2021 Jun 4];29(6):795–808. Available from: https://academic.oup.com/heapol/article/29/6/795/576992. 10.1093/heapol/czt064 PMC4153302 24097794

[7] Organização Mundial da Saúde. AFR/RC67/13 15 de Junho de 2017 COMITÉ REGIONAL PARA A ÁFRICA Sexagésima sétima sessão [Internet]. 2017. Available from: https://www.afro.who.int/sites/default/files/2017-08/AFR-RC67-13 Relatório sobre o Estado de Implementação da década de Acção para a Segurança Rodoviária na Região Africana.pdf.

[8] World Health Organization. Making choices in health: WHO guide to cost-effectiveness analysis [Internet]. Hutubessy RCW, Baltussen R, Tan Torres-Edejer T, Evans DB, editors. Health systems performance assessment: debates, methods and empiricism. Geneva: WHO Editions. Geneva: World Health Organization; 2003. 823–835 p. Available from: http://books.google.com/books?hl=en&lr=&id=_HloWI6HXbcC&oi=fnd&pg=PR9&dq=edejer+baltussen&ots=hgTnNiuW9x&sig=ffrEjZ6TyLp1EM1y39gYfpVBno8.

[9] Rumisha SF, George J, Bwana VM, Mboera LEG. Years of potential life lost and productivity costs due to premature mortality from six priority diseases in Tanzania, 2006–2015. Fischer F, editor. PLoS One [Internet]. 2020 Jun 9 [cited 2021 Feb 18];15(6):e0234300. Available from: https://dx.plos.org/10.1371/journal.pone.0234300. doi: 10.1371/journal.pone.0234300 PMC7282655 32516340

[10] Najafi F, Karami-Matin B, Rezaei S, Khosravi A, Soofi M. Productivity costs and years of potential life lost associated with five leading causes of death: Evidence from Iran (2006–2010). Med J Islam Repub Iran [Internet]. 2016 [cited 2021 Feb 18];30(1):412. Available from: http://mjiri.iums.ac.ir. doi: 10.1136/bmjopen-2014-005386 28210577 PMC5307621

[11] Souza VMM de, Arsky M de LNS, Castro APB de, Araujo WN de, Mendes De Souza VM, Peres Barbosa De Castro A. Anos potenciais de vida perdidos e custos hospitalares da leptospirose no Brasil. Rev Saúde Pública [Internet]. 2011 Dec [cited 2019 Nov 13];45(6):1001–8. Available from: www.scielo.br/rsp. 21953079 10.1590/s0034-89102011005000070

[12] Ministério da Saúde e da Segurança Social da República de Cabo Verde. RELATÓRIO ESTATÍSTICO 2018 [Internet]. Praia; 2019 [cited 2020 Sep 22]. Available from: https://www.insp.gov.cv/index.php/observatorio-saude/relatorios-estatisticos/289-relatorio-estatistico-2018-final/file.

[13] Ministério da Saúde de Cabo Verde. Plano Nacional Desenvolvimento Sanitário 2012–2016 [Internet]. 1st ed. Ministério da Saúde CV, editor. Vol. I. Praia: Ministério da Saúde, Cabo Verde; 2012. 92 p. Available from: http://www.minsaude.gov.cv/.

[14] Regional-West 2 Department. CAPE VERDE A SUCCESS STORY Regional-West 2 Department (ORWB) [Internet]. 2012 [cited 2021 Mar 30]. Available from: https://www.afdb.org/sites/default/files/documents/projects-and-operations/cape_verde_-_a_success_story.pdf.

[15] Carvalho JMC de. REPÚBLICA DE CABO VERDE PROGRAMA DAS NAÇÕES UNIDAS PARA O DESENVOLVIMENTO RELATÓRIO NACIONAL Preparação da IIIa Conferência Internacional sobre Desenvolvimento Sustentável nos Pequenos Estados Insulares em Desenvolvimento [Internet]. Praia; 2013 [cited 2022 Oct 7]. Available from: https://sustainabledevelopment.un.org/content/documents/1059297CaboVerde_Report.PTversion.pdf.

[16] Instituto Nacional de Estatistica de Cabo Verde. Projecções demográficas da população por concelho e faixa etária, 2010–2030—INE [Internet]. PROJECÇÕES DEMOGRÁFICAS. 2017 [cited 2021 Feb 1]. Available from: http://ine.cv/quadros/resumo-das-projeccoes-demograficas-da-populacao-concelho-2010-2030/.

[17] World Health Organization (WHO). ICD-10 Version:2016 [Internet]. International Statistical Classification of Diseases and Related Health Problems 10th Revision (ICD-10)-WHO Version for;2016. 2016 [cited 2022 Apr 6]. Available from: https://icd.who.int/browse10/2016/en#/XX.

[18] Mendeley. Free Reference Manager & Citation Generator—Mendeley [Internet]. Mendeley Desktop. 2020 [cited 2021 Feb 26]. Available from: https://www.mendeley.com/reference-management/mendeley-desktop.

[19] Zuraik C, Sampalis J. Epidemiology of Traumatic Injuries at an Urban Hospital in Port-au-Prince, Haiti. World J Surg. 2017 Nov 1;41(11):2674–80. doi: 10.1007/s00268-017-4088-2 28681141

[20] Gardener JW, Stanborn JS. Years of Potential Life Lost (YPLL) __What Does it Measure. Epidemiology [Internet]. 1990;1(4):322–9. Available from: https://journals.lww.com/epidem/Abstract/1990/07000/Years_of_Potential_Life_Lost__YPLL__What_Does_it.12.aspx. 2083312 10.1097/00001648-199007000-00012

[21] The World Bank. Life expectancy at birth, total (years)—Cabo Verde | Data [Internet]. Life expectancy at birth, total (years)—Cabo Verde | Data. 2019 [cited 2021 Feb 7]. Available from: https://data.worldbank.org/indicator/SP.DYN.LE00.IN?locations=CV.

[22] Instituto Nacional de Estatistica Cabo Verde. Anuario Estatistico 2018 [Internet]. Praia; 2020 [cited 2021 Feb 19]. Available from: http://ine.cv/wp-content/uploads/2020/10/aecv-2018.pdf.

[23] Banco de Cabo Verde. RELATÓRIO ANUAL 2018 [Internet]. Praia; 2019 [cited 2021 Mar 13]. Available from: https://www.bcv.cv/en/Estatisticas/PublicacoeseIntervencoes/Relatorios/RelatorioAnual/Documents/AnnualReport2017Inglês.pdf

[24] World Health Organization Regional Office for Europe. Methodological approaches for cost-effectiveness and cost-utility analysis of injury prevention measures [Internet]. 1st ed. Polinder S, Toet H, Panneman M, Van Beeck E, editors. Copenhagen; 2011 [cited 2021 Apr 24]. Available from: http://www.euro.who.int/pubrequest.

[25] Bhalla Kavi, Harrison James, Shahraz Saeid, Abraham Jerry, Bartels David, Yeh Pon-Hsiu, et al. BURDEN OF ROAD INJURIES IN SUB-SAHARAN AFRICA. 2014;6(2):103. Available from: http://pubdocs.worldbank.org/en/356861434469785833/Road-Safety-Burden-of-Injuries-in-Africa.pdf.

[26] Matzopoulos R, Prinsloo M, Pillay-Van Wyk V, Gwebushe N, Mathews S, Martin LJ, et al. Injury-related mortality in south africa: A retrospective descriptive study of postmortem investigations. Bull World Health Organ. 2015;93(5):303–13. doi: 10.2471/BLT.14.145771 26229201 PMC4431514

[27] Department of Statistics South Africa [Stats SA]. Mortality and causes of death in South Africa, 2017: Findings from death notification [Internet]. Statistical release. Pretoria; 2020. Available from: www.statssa.gov.za,info@statssa.gov.za,Tel+27123108911.

[28] Chisumpa VH, Odimegwu CO. Decomposition of age- and cause-specific adult mortality contributions to the gender gap in life expectancy from census and survey data in Zambia. SSM—Popul Heal [Internet]. 2018 Aug [cited 2019 Sep 20];5:218–26. Available from: http://www.ncbi.nlm.nih.gov/pubmed/30094317. 10.1016/j.ssmph.2018.07.003 PMC6077128 30094317

[29] Sorenson SB. Gender disparities in injury mortality: consistent, persistent, and larger than you’d think. Am J Public Health [Internet]. 2011 Dec 28 [cited 2019 Sep 20];101 Suppl(S1):S353. Available from: http://ajph.aphapublications.org/doi/10.2105/AJPH.2010.300029. 21778511 10.2105/AJPH.2010.300029 PMC3222499

[30] Tyson AF, Varela C, Cairns BA, Charles AG. Hospital mortality following trauma: An analysis of a hospital-based injury surveillance registry in sub-Saharan Africa. J Surg Educ [Internet]. 2015;72(4):e66–72. Available from: doi: 10.1016/j.jsurg.2014.09.010 25451718

[31] Nobre DM., Cavalcante DH., Coelho MP. Mortalidade, morbidade hospitalar e atendimentos de emergência por causas externas no Brasil [Internet]. Mortalidade, morbidade hospitalar e atendimentos de emergência por causas externas no Brasil. [CAJAZEIRAS]: Monografia de Bacharel em Medicina, Universidade Federal de Campina Grande, campus Cajazeiras,; 2016. Available from: http://dspace.sti.ufcg.edu.br:8080/jspui/handle/riufcg/8212.

[32] Pillay-van Wyk V, Msemburi W, Laubscher R, Dorrington RE, Groenewald P, Glass T, et al. Mortality trends and differentials in South Africa from 1997 to 2012: second National Burden of Disease Study. Lancet Glob Heal [Internet]. 2016 Sep 1 [cited 2021 Mar 22];4(9):e642–53. Available from: 10.1016/S2214-109X(16)30113-9. 27539806

[33] Malta DC, De Souza Minayo MC, Filho AMS, Da Silva MMA, De Mesquita Silva Montenegro M, Ladeira RM, et al. Mortalidade e anos de vida perdidos por violências interpessoais e autoprovocadas no Brasil e Estados: Análise das estimativas do Estudo Carga Global de Doença, 1990 e 2015. Rev Bras Epidemiol [Internet]. 2017 [cited 2021 Feb 16];20(1):142–56. Available from: http://www.scielo.br/scielo.php?script=sci_arttext&pid=S1415-790X2017000500142&lng=en&nrm=iso&tlng=en. 10.1590/1980-5497201700050012 28658379

[34] Xing X-Y, Wang P, Xu Z, He Q, Li R, Chen Y-J, et al. Mortality and Disease Burden of Injuries from 2008 to 2017 in Anhui Province, China. Biomed Res Int [Internet]. 2020 [cited 2021 Jul 15];2020:1–10. Available from: https://pubmed.ncbi.nlm.nih.gov/32382567/. doi: 10.1155/2020/7303897 32382567 PMC7193292

[35] Corassa RB, Falci DM, Gontijo CF, Machado GVC, Alves PAB. Evolução da mortalidade por causas externas em Diamantina (MG), 2001 a 2012. Cad Saúde Coletiva [Internet]. 2017;25(3):302–14. Available from: http://www.scielo.br/scielo.php?script=sci_arttext&pid=S1414-462X2017000300302&lng=pt&tlng=pt.

[36] Haagsma JA, Graetz N, Bolliger I, Naghavi M, Higashi H, Mullany EC, et al. The global burden of injury: Incidence, mortality, disability-adjusted life years and time trends from the global burden of disease study 2013. Inj Prev [Internet]. 2016;22(1):3–18. Available from: https://injuryprevention.bmj.com/content/22/1/3. doi: 10.1136/injuryprev-2015-041616 26635210 PMC4752630

[37] Nacional Policia. Crimes Contra Pessoas 2017–2018 [Internet]. Crimes Contra Pessoas 2017–2018. 2018 [cited 2021 Aug 14]. Available from: https://www.policianacional.cv/index.php/component/docman/cat_view/86-estatistica-2017-2018?Itemid=1.

[38] Republica de Cabo Verde. Decreto-Legislativo no 1/2007: Introduz alterações ao Decreto-Legislativo no 4/2005, de 26 de Setembro que aprovou o Código de Estrada. [Internet]. Republica de Cabo Verde, 1/2007 Praia, Republica de Cabo Verde: Boletim Oficial I serie no 17 Sexta-feira, 11 de Maio de 2007; 2007 p. 1–47. Available from: https://portondinosilhas.gov.cv/images/igrp-portal/img/documentos/49BD996914D979F6E053E700040AAE7E.pdf.

[39] Republica de Cabo Verde. Decreto-Lei n.o 84/VII/11, de 10 de Janeiro, que estabelece as medidas destinadas a prevenir e reprimir o crime de violência baseada no género (VBG), [Internet]. Republica de Cabo Verde, 8/2014 Praia, Republica de Cabo Verde: Boletim Oficial da Republica de Cabo Verde, I Serie–no 08–27 de janeiro de 2015; 2015 p. 1–16. Available from: http://portais.parlamento.cv/rmpcv/legislacao/Decreto-Lei que regulamenta a Lei VBG.pdf.

[40] Organização Mundial de Saúde—Instituto Cabo-verdiano para a Igualdade e Equidade de Género. Manual de Procedimentos para Serviços Profissionais de Saúde: Prevenção e atendimento às vítimas de violência baseada no género [Internet]. MANUAL DE PROCEDIMENTOS PARA SERVIҪOS E PROFISSIONAIS:DE SAÚDE Prevenção e atendimento às vítimas de VBG. Praia; 2015 [cited 2019 Nov 20]. Available from: https://www.minsaude.gov.cv/index.php/documentosite/451-manual-de-procedimentos-para-servicos-e-profissionais-de-saude-prevencao-e-atendimento-as-vitimas-de-violencia-baseada-no-genero-vbg/file.

[41] Abio A, Bovet P, Didon J, Bärnighausen T, Shaikh MA, Posti JP, et al. Trends in mortality from external causes in the Republic of Seychelles between 1989 and 2018. Sci Reports 2020 101 [Internet]. 2020 Dec 17 [cited 2021 Dec 27];10(1):1–11. Available from: https://www.nature.com/articles/s41598-020-79228-8. doi: 10.1038/s41598-020-79228-8 33335193 PMC7746745

[42] Saunders CJ, Sewduth D, Naidoo N. Keeping our heads above water: A systematic review of fatal drowning in South Africa. South African Med J [Internet]. 2017 Dec 13;108(1):61. Available from: http://www.samj.org.za/index.php/samj/article/view/12185. 10.7196/SAMJ.2017.v108i1.11090 29262981

[43] Ministério da Saúde. POLÍTICA, NORMAS E PROCEDIMENTOS DOS SERVIÇOS DE SAÚDE SEXUAL E REPRODUTIVA. Vol. 1, Ilha Revista de Antropologia. Praia: Ministério da Saúde, Cabo Verde; 2021. p. 13–20.

[44] Delgado M. Transição Epidemiológica/Perfil de Mortalidade da População de São Vicente, Cabo Verde, no Ano de 2010 [Internet]. 2013. Available from: http://193.136.21.50/handle/10961/4713.

[45] Rajabali F, Beaulieu E, Smith J, Pike I. The economic burden of injuries in British Columbia: Applying evidence to practice. B C Med J. 2018;60(7):358–64.

[46] World Health Organization (WHO). A heavy burden: the productivity cost of illness in Africa [Internet]. Brazaville; 2019 [cited 2021 Feb 18]. Available from: http://apps.who.int/iris.

[47] Šemerl JŠ, Šešok J. Years of potential life lost and valued years of potential life lost in assessing premature mortality in Slovenia. Croat Med J. 2002;43(4):439–45. 12187522

[48] Davey S, Bulat E, Massawe H, Pallangyo A, Premkumar A, Sheth N. The Economic Burden of Non-fatal Musculoskeletal Injuries in Northeastern Tanzania. Ann Glob Heal [Internet]. 2019 [cited 2021 Jul 15];85(1). Available from: https://pubmed.ncbi.nlm.nih.gov/30873794/. doi: 10.5334/aogh.1355 30873794 PMC6997525

[49] Pike J, Grosse SD. Friction cost estimates of productivity costs in cost-of-illnessstudies in comparison with human capital estimates: a review. Appl Health Econ Health Policy [Internet]. 2018 Dec 1 [cited 2021 Aug 27];16(6):765. Available from: /pmc/articles/PMC6467569/. doi: 10.1007/s40258-018-0416-4 30094591 PMC6467569

[50] Koopmanschap MA, Rutten FFH, Martin Van Ineveld B, Van Roijen L. The friction cost method for measuring indirect costs of disease. J Health Econ [Internet]. 1995 [cited 2021 Aug 27];14:171–89. Available from: https://www.sciencedirect.com/science/article/abs/pii/0167629694000445?via%3Dihub. doi: 10.1016/0167-6296(94)00044-5 10154656

